# Biotic interactions are an unexpected yet critical control on the complexity of an abiotically driven polar ecosystem

**DOI:** 10.1038/s42003-018-0274-5

**Published:** 2019-02-15

**Authors:** Charles K. Lee, Daniel C. Laughlin, Eric M. Bottos, Tancredi Caruso, Kurt Joy, John E. Barrett, Lars Brabyn, Uffe N. Nielsen, Byron J. Adams, Diana H. Wall, David W. Hopkins, Stephen B. Pointing, Ian R. McDonald, Don A. Cowan, Jonathan C. Banks, Glen A. Stichbury, Irfon Jones, Peyman Zawar-Reza, Marwan Katurji, Ian D. Hogg, Ashley D. Sparrow, Bryan C. Storey, T. G. Allan Green, S. Craig Cary

**Affiliations:** 1 School of Science, University of Waikato, Hamilton, 3240 New Zealand; 2 International Centre for Terrestrial Antarctic Research, University of Waikato, Hamilton, 3240 New Zealand; 3 School of Biological Sciences and Institute for Global Food Security, Queen’s University Belfast, Belfast, BT7 1NN UK; 4 Department of Biological Sciences, Virginia Tech, Blacksburg, VA 24061 USA; 5 School of Social Sciences, University of Waikato, Hamilton, 3240 New Zealand; 6 Hawkesbury Institute for the Environment, Western Sydney University, Penrith, NSW 2751 Australia; 7 Department of Biology, Evolutionary Ecology Laboratories, and Monte L. Bean Museum, Brigham Young University, Provo, UT 84602 USA; 8 Department of Biology & School of Global Environmental Sustainability, Colorado State University, Fort Collins, CO 80523 USA; 9 SRUC – Scotland’s Rural College, Edinburgh, EH9 3JG UK; 10 Yale-NUS College and Department of Biological Sciences, National University of Singapore, Singapore, 138527 Singapore; 11 Centre for Microbial Ecology and Genomics, Department of Biochemistry, Genetics and Microbiology, University of Pretoria, Pretoria, 0002 South Africa; 12 Environmental Research Institute, University of Waikato, Hamilton, 3240 New Zealand; 13 Gateway Antarctica, University of Canterbury, Christchurch, 8041 New Zealand; 14 Centre for Atmospheric Research, Department of Geography, University of Canterbury, Christchurch, 8041 New Zealand; 15 CSIRO Ecosystem Sciences, Alice Springs, NT 0870 Australia; 16 Departamento de Biología Vegetal II, Facultad de Farmacia, Universidad Complutense de Madrid, Madrid, 28040 Spain; 17 College of Earth and Ocean Sciences, University of Delaware, Newark, DE 19958 USA; 18 Present Address: Department of Botany, University of Wyoming, Laramie, WY 82071 USA; 19 Present Address: Department of Biology, Thompson Rivers University, Kamloops, BC V2C 0C8 Canada; 20 Present Address: Cawthron Institute, Nelson, 7010 New Zealand; 21 Present Address: Polar Knowledge Canada, Canadian High Arctic Research Station, Cambridge, Bay, X0B 0C0 Nunavut Canada

## Abstract

Abiotic and biotic factors control ecosystem biodiversity, but their relative contributions remain unclear. The ultraoligotrophic ecosystem of the Antarctic Dry Valleys, a simple yet highly heterogeneous ecosystem, is a natural laboratory well-suited for resolving the abiotic and biotic controls of community structure. We undertook a multidisciplinary investigation to capture ecologically relevant biotic and abiotic attributes of more than 500 sites in the Dry Valleys, encompassing observed landscape heterogeneities across more than 200 km^2^. Using richness of autotrophic and heterotrophic taxa as a proxy for functional complexity, we linked measured variables in a parsimonious yet comprehensive structural equation model that explained significant variations in biological complexity and identified landscape-scale and fine-scale abiotic factors as the primary drivers of diversity. However, the inclusion of linkages among functional groups was essential for constructing the best-fitting model. Our findings support the notion that biotic interactions make crucial contributions even in an extremely simple ecosystem.

## Abstract (web summary)

Charles Lee, Daniel Laughlin et al. use structural equation modeling to analyze ecological data from more than 500 sites in the Antarctic Dry Valleys. They find that although abiotic factors are the primary drivers of biodiversity variation, biotic interactions are needed to explain the data fully and may play previously underestimated roles.

## Introduction

Understanding how ecosystems self-organize at landscape scales has long been a formidable challenge in ecology^1,2^ since the trophic complexity of most ecosystems obscures the relative contributions of the biotic and abiotic factors regulating biological diversity^3–6^. Given the fundamental effects of biodiversity on ecosystem function^7^, a critical task is to resolve the relative importance of three sets of ecological factors that drive community structure: abiotic environmental filtering, dispersal limitation in space, and biotic interactions (e.g., competition, mutualism, and trophic relationships)^2,8^. Thorough and spatially explicit descriptions of these ecosystem drivers are required for this task, but the complexity of most ecosystems creates enormous logistical obstacles.

Biotic interactions, including those among higher eukaryotes and those between higher eukaryotes and microorganisms, have long been recognized as important drivers of ecosystem structure and function^6,9^. However, attempts to capture biotic interactions at the ecosystem level have often been restricted both by sampling approaches and/or the expertise of individual investigators^10^, and studies have largely focused on testing hypotheses associated with pre-identified biotic interactions or ecosystem components^6,11–15^. A comprehensive investigation of abiotic and biotic interactions within an ecosystem therefore requires a sampling design that is consistent across all major biological groups present. It also requires an explicitly interdisciplinary and comprehensive approach for data collection and analysis of both abiotic and biotic variables.

For microorganisms (bacteria, archaea, and unicellular fungi), culture-independent characterization using molecular genetic techniques is widely recognized as the most consistent and sensitive approach^16^, whereas conventional surveys remain the most reliable and practical approach for larger invertebrates and higher animals and plants in terrestrial environments. For abiotic variables and some major macroecological features (e.g., primary productivity), geographic information system (GIS) has become an essential tool for collecting information across spatial scales. The integration of GIS and remote sensing technologies (e.g., satellites) can provide spatially explicit environmental information for entire geographic regions^17,18^. Specifically, the availability of high-resolution data layers from sources such as the Landsat 7 and MODIS satellites facilitates complete and consistent descriptions of environmental conditions (e.g., surface temperature and snow coverage) at the landscape scale^18,19^. Using these descriptions in conjunction with information on bedrock geology and geomorphology, it is now feasible to carry out systematic landscape-scale surveys that capture heterogeneities in abiotic conditions within an ecosystem. Additionally, all information collected within a GIS-enabled sampling framework is spatially explicit and enables thorough examinations of dispersal limitation effects across multiple spatial scales.

**Fig. 1:**
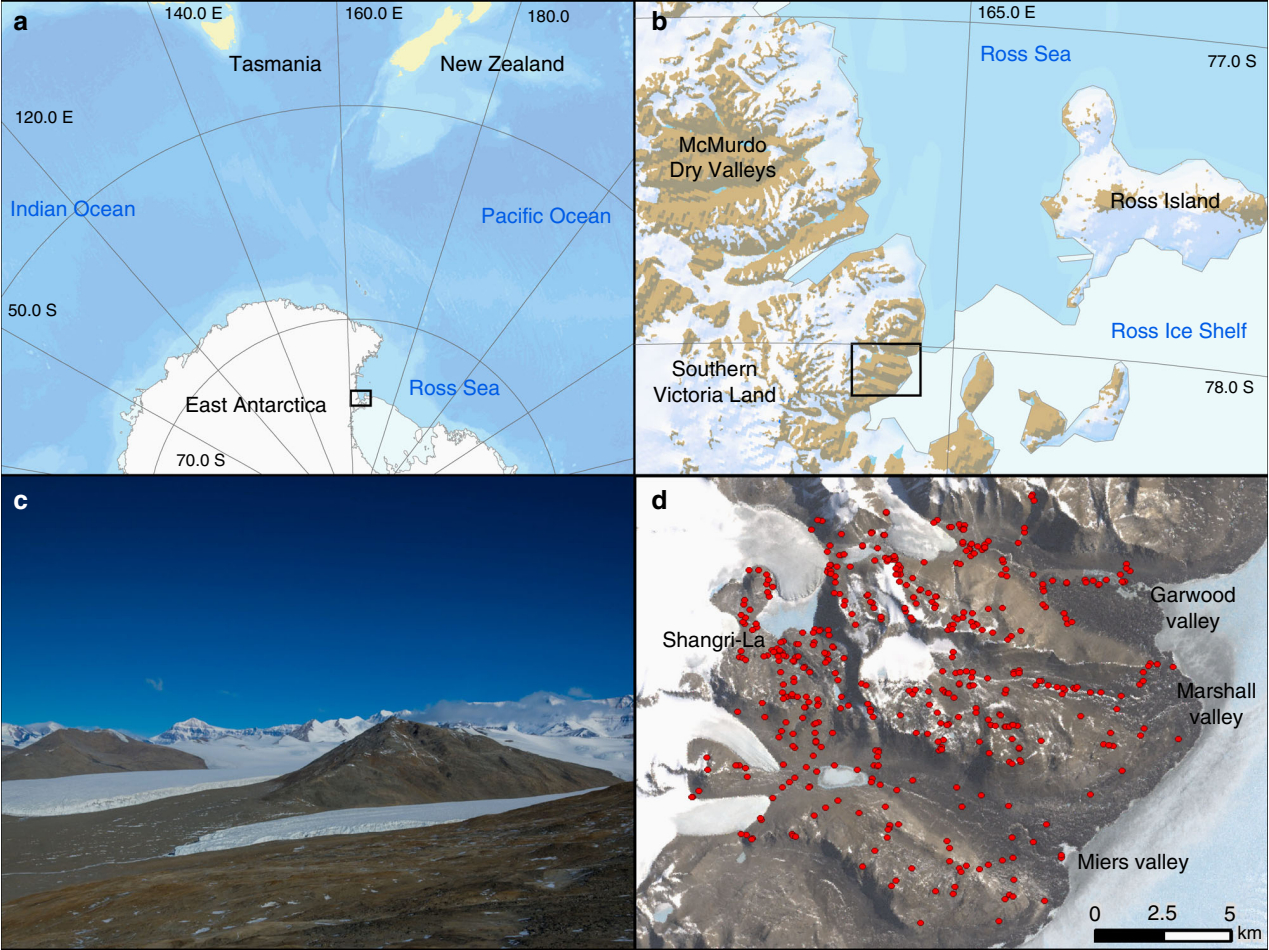
Maps. **a** Southern Victoria Land (denoted by black rectangle) relative to East Antarctica and New Zealand (Image Credit: the World Topographic Map, ArcGIS Online, Esri); **b** the McMurdo Dry Valleys (nzTABS study area denoted by black rectangle) relative to southern Victoria Land; **c** westward view of the Miers Valley toward the Royal Society Range; and **d** the nzTABS study area, including Miers, Marshall, and Garwood Valleys (sampling sites denoted by red dots) (Image Credit: the Landsat Image Mosaic of Antarctica [LIMA] Project)

Despite the advances in methodologies, disentangling the relative roles of abiotic and biotic controls on the complexity in terrestrial ecosystems is still a major challenge in ecology. We propose that extreme ecosystems offer a natural laboratory to reduce this complexity while representing its major features^20^. Here, we offer an analysis of the controls on the biological complexity of a region of the McMurdo Dry Valleys of Antarctica. Located between the Polar Plateau and the Ross Sea (Fig. 1), the McMurdo Dry Valleys (hereinafter the Dry Valleys) are the largest contiguous ice-free area on the Antarctic continent and subject to some of the most extreme conditions of any terrestrial habitat on Earth^21^, which severely constrain the range of biota present^22,23^. Vascular plants and vertebrates are entirely absent, and soils are predominantly ultraoligotrophic, hyperarid, and often hypersaline^21,22^. Consequently, abiotic factors are widely regarded as the primary force shaping the ecology of Dry Valley soils^20,21,24–27^. The extreme environmental conditions and lack of evidence for critical biotic interactions have made ecologists hypothesize that these ecosystems are fundamentally constrained by abiotic factors and host some of the simplest trophic structures on Earth^21,23,24,28,29^ (Supplementary Figure 1). These unique characteristics make the Dry Valleys a model system for resolving the roles of abiotic and biotic factors that shape community structure.

In this study, we analyzed data collected by the New Zealand Terrestrial Antarctic Biocomplexity Survey (nzTABS, https://ictar.aq/nztabs-science/), which was initiated during the International Polar Year 2007–2008. This project draws on a wide range of international expertise to profile the biology, geochemistry, geology, and climate of the Dry Valleys, and has completed a spatially and biologically comprehensive landscape-scale survey that aims to resolve the biotic and abiotic control of ecosystem complexity. We then used structural equation modelling (SEM) to analyze the comprehensive collection of geological, geographical, geochemical, hydrological, and biological variables measured systematically across three Dry Valleys of Antarctica.

Structural equation modelling (SEM), which is built on path analysis and factor analysis, is one of the most useful statistical approaches to disentangle numerous factors of influence^30^ and develop deeper causal understanding from observational data^31^. Users of SEM translate theoretical frameworks (informed by knowledge of the ecosystem) into explicit multivariate hypotheses, and the SEM is used to evaluate whether the theory is consistent with empirical data^30,32^. A robust SEM allows researchers to quantitatively assess the relative importance of ecological drivers^32,33^ and is thus well suited for predicting ecosystem responses to global change^1,33^.

In addition to challenging climatic conditions and hyperaridity^21^, biotic interactions in Dry Valley soils are thought to be limited by very low biomass and patchy distribution of phototrophic communities^28^. Temperature and biologically available water have been proposed as the primary determinants of species occurrence^21,34^, but the lack of studies explicitly addressing biotic interactions in the Dry Valley ecosystems means that the role of biotic interactions is largely unknown (if present at all)^28^. Therefore, our primary hypothesis was that abiotic environmental filters are the major control on the biodiversity of this environmentally extreme and simple terrestrial ecosystem, and the structure of our SEM mostly reflected this notion. However, we found that linkages between different groups of biota and the groups’ discrete spatial patterns (i.e., independent of the effects of spatial variation in abiotic factors) had to be included to obtain the best-fitting SEM. Overall, these findings indicate that biotic interactions make crucial contributions even in an extremely simple ecosystem.

## Results

### Correlations and nestedness of measured variables

Our data indicated that richness (see Methods for definitions) was related to community composition in each of the three groups of organisms examined (i.e., multicellular taxa, cyanobacteria, and fungi), which was to be expected given the low species richness of each group in the analyzed system. Multicellular taxon communities were significantly nested (nestedness temperature = 17.56, *P* = 0.010), and the richness of multicellular taxa was strongly correlated (*r* = −0.99, *P* = 0.001) with the first NMS axis of multicellular community composition. Nematodes were by far the most frequently observed organism, occurring in 80% of the sampled tiles, followed by hypoliths (40%), rotifers (32%), lichens (25%), tardigrades (23%), springtails (19%), cyanobacterial mats (15%), mites (12%), and mosses (11%). Cyanobacterial communities were significantly nested (nestedness temperature = 2.01, *P* = 0.010), and cyanobacterial richness was correlated with both NMS axes (axis 1: *r* = −0.75, axis 2: *r* = −0.66, *P* = 0.001) derived from the cyanobacterial community matrix. Fungal communities were also significantly nested (nestedness temperature = 2.61, *P* = 0.010), and fungal richness was correlated with both NMS axes (axis 1: *r* = 0.61, axis 2: *r* = −0.79, *P* = 0.003) derived from the fungal community matrix.

### Structural equation models

**Fig. 2:**
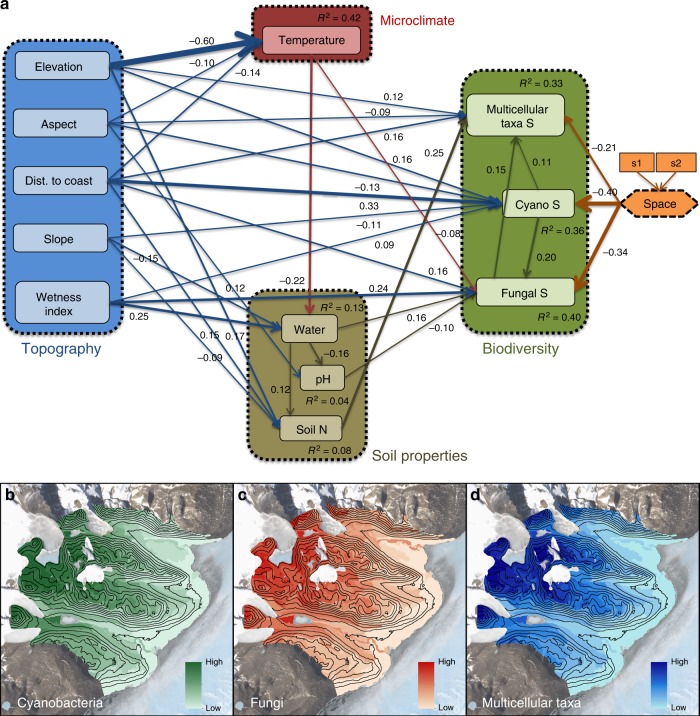
Final structural equation model and predicted richness for three biotic groups. **a** Final structural equation model (CFI = 0.996, *χ*^2^ = 45.018, df = 35, *P* = 0.1196) with standardized path coefficients (all paths significant, see the Mplus code in Supplementary Data 4). “S” represents the richness (and composition) of multicellular taxon and microbial assemblages. “Space” represents environmentally independent spatial variables. Cyanobacterial richness was positively correlated with elevation, distance to the coast, and the wetness index; negatively correlated with aspect (degrees from north) and slope; and strongly related to spatial covariates. Fungal richness was positively correlated with distance from the coast, soil water content, and cyanobacteria richness; negatively correlated with pH and temperature; and strongly related to spatial covariates. Richness of multicellular taxa was positively correlated with cyanobacterial richness, fungal richness, soil nitrogen, distance to the coast, and elevation; negatively correlated with aspect; and less strongly related to spatial covariates. Higher surface temperatures were associated with lower soil water content; and **b**–**d** predictions of cyanobacterial, fungal, and multicellular taxon richness, respectively, across the landscape

The initial *a priori* SEM (Supplementary Figure 2) did not fit the data well (Comparative Fit Index [CFI] = 0.706, *χ*^2^ = 394.397, df = 43, *P* < 0.0001). This initial model only included pathways that were well supported by previous empirical studies at the time the model was fit. This initial result made it clear that our *a priori* expectations were missing important relationships within the ecosystem that were not well known in the literature. Consequently, to obtain a model with an implied covariance structure that matched the observed data well, we made two modifications. First, we identified missing pathways that contributed to poor model-fit by inspecting the residual covariance matrix and added as few of these theoretically plausible pathways as possible (e.g., direct pathways from abiotic variables to biotic variables). Second, we removed non-significant pathways. The final model (Fig. 2a) fits the data well (CFI = 0.996, *χ*^2^ = 45.018, df = 35, *P* = 0.1196) and represents the most parsimonious model possible. Each pathway in the model is significant (the standardized path coefficients can be interpreted as partial correlation coefficients). The model explains between 30 and 40% of the variance in the richness variables (i.e., Multicellular Taxa S, Cyano S, and Fungal S), which are strongly correlated with community composition, as described above. Importantly, soil properties do not clearly mediate the effects of topography and climate on biotic diversity, and topographic and climate variables have many important direct pathways to biota. Attempts to trim the model by removing select pathways substantially reduced the goodness-of-fit of the model, so each pathway is important. This model is particularly valuable and robust because it explicitly accounts for spatial patterns that do not depend on environmental variables (see Methods). For example, some areas could be richer in species simply because they are located in regions that receive a higher supply of immigrants supporting local populations even when conditions are not favorable. The ‘total effects’ (i.e., sums of direct and indirect effects) of each environmental variable on each biotic variable (Multicellular Taxa S, Cyano S, and Fungal S) indicate that elevation, slope, aspect, distance to coast, and wetness index all have significant total effects on richness and composition of multicellular taxon and microbial assemblages (Supplementary Table 1).

### Importance of biotic interactions

It is also important to note that the positive links among the richness of multicellular taxon and microbial assemblages were essential to the model; removing these pathways yielded very poor model-fit indices. The chosen directions of these pathways were guided by both empirical data and theory. There were eight possible combinations of directed paths among three variables, and after arriving at the final model (Fig. 2a), we tested all eight combinations to evaluate the sensitivity to the directions of these pathways. Four of these eight models yielded poor-fitting models (*P* < 0.05, specific models not shown), and each of these poor models included a pathway from multicellular taxa to fungi, which is strong evidence against that particular pathway. However, the other four models were indistinguishable from a model-fitting perspective (all *P* > 0.05, specific models not shown), and so we relied on theory to specify the direction of these pathways. Ecological theory supports pathways from cyanobacterial richness to fungal richness and from cyanobacterial richness to multicellular taxon richness, given the foundational contribution of these autotrophic single-celled organisms to this extreme ecosystem. Importantly, the positive covariance among the biota is not simply due to similar responses to abiotic conditions because each group responds individually to the sets of abiotic variables in the model (Fig. 2a). This implies that processes other than abiotic filtering drive the positive covariance among the three groups of biota.

### Relative contributions of abiotic and biotic factors

Overall, environmental filtering imposed the strongest net effects on biotic richness (Table 1). Spatial processes were the second most important set of richness drivers, with nearly the same magnitude of effect as environmental filtering for cyanobacteria (Table 1). Biotic interactions were important in determining fungal and multicellular taxon richness, and their impact on multicellular taxa was comparable to that of spatial processes (Table 1). Finally, the SEM was used to generate spatially explicit predictions of biodiversity across the study area (Fig. 2b–d) to demonstrate its potential as a tool for understanding the spatial heterogeneity of soil biota in the Dry Valleys for both scientific investigation and environmental management.

**Table 1:** Net effects of various parameters on biological richness

	Abiotic	Spatial	Biotic
Cyanobacteria	0.45	0.40	0
Fungi	0.42	0.34	0.20
Multicellular Taxa	0.39	0.21	0.21

Net effects of abiotic environmental filters, spatial processes, and biotic interactions on cyanobacterial, fungal, and multicellular taxon richness. Effects were calculated using composite variables within the SEM and represent the absolute standardized path coefficients (ranging from 0 to 1).

## Discussion

Earlier investigators had suggested that the species richness of Antarctic terrestrial vegetation south of 72°S^35^ and the structure of Dry Valley invertebrate communities^26,36^ are determined by local conditions. Our data and model show the prominence of abiotic drivers (in particular total soil N, soil wetness index, elevation, and distance to the coast) and support the general view that abiotic factors are the most important ecological filter in extreme environments^2,20^. However, there is a notable and large amount of variance in the species richness of major functional groups and their reciprocal correlations that is not accounted for by abiotic factors. Notably, soil ATP level (a proxy for biomass) was not significantly correlated with any of the other variables measured and removed from the final model (Fig. 2a). In the absence of other practical measures of biotic variables, we believe that species richness (which is significantly correlated with composition, see Methods) effectively captures biotic variables within this study.

Our model shows that the spatial autocorrelation vectors (i.e., independent of variation in abiotic factors) are the second strongest correlate of richness (Table 1). The spatial patterns accounted for by these autocorrelation vectors can be caused by both unmeasured biological processes (e.g., dispersal limitation) and legacy effects (e.g., historic distribution of glaciers and pro-glacial lakes)^20^. It is very unlikely that these spatial patterns are caused by major unmeasured environmental variables, given the quantity and quality of the environmental measurements collected in this study. It is possible that an unmeasured soil attribute (e.g., soil bulk density, water-holding capacity) could account for some of the patterns observed. However, given the variety of biotic variables captured in this study, it is unlikely that any single unmeasured abiotic variable will show strong correlation with measured biotic influences. Legacy effects are important in establishing and maintaining ice-free refugia for terrestrial biota^20,37^, and the importance of the spatial vectors in the SEM thus potentially supports a role for legacies linked to glacial geomorphology in shaping distributions of biota in the Dry Valleys^36,38^.

Our model also shows that, besides spatial vectors and abiotic factors, a notable amount of variance in the species richness of each group is explained by correlations between biotic groups. As we further explain below, we hypothesize that these correlations reflect variation in the biological complexity of the ecosystem. Specifically, our model highlights some of the major linkages in the Dry Valleys ecosystem: in this system, cyanobacteria provide the energetic foundation of food webs, and their species richness does not appear to be influenced by the richness of fungi and multicellular taxa (Table 1). However, fungal richness was highest where cyanobacterial richness was high, and multicellular taxon richness was highest where both cyanobacterial and fungal richness was high (Fig. 2b–d), highlighting the fundamental importance of autotrophic cyanobacteria as the primary producers in this extreme ecosystem. We are confident that the positive covariance among the groups of organisms considered here is not confounded by covariation with abiotic conditions because the model explicitly allowed each group to respond to a unique combination of abiotic variables. Conversely, the set of correlations that link the species richness of the three groups are essential to the fit of the model; removing them from the model produces models that fit the data very poorly.

The Dry Valleys are arguably the simplest large-scale (4500 km^2^ of ice-free area^39^) ecosystem on the Earth. Therefore, a small increase in the richness of any of the three major groups in this system may imply a disproportionate increase in the biotic complexity of the system because every added species can bring in a new set of interactions between the three functional groups. This is consistent with the trophic theory of island biogeography, which is particularly relevant to systems such as the Dry Valleys because they experience dispersal limitation and disconnection between local communities^37,40–43^. Specifically, trophic constraints (i.e., species need their resource to establish successfully) alter immigration and extinction dynamics, which ultimately determine species richness. In the Dry Valleys, food webs are particularly isolated compared to other soil food webs, which should reduce the recruitment of lower trophic level species for their consumers. The low connectivity of the food webs in the Dry Valleys is thus expected to translate into lower immigration and higher extinction rates. This is expected to create high spatial and temporal variability in species composition and richness, and contributes to food webs dominated by generalist primary consumers with very few secondary consumers^40,41^. Overall, the patterns of species richness we observed are consistent with these dynamics and suggest that the diversity of the primary producers plays a central role in driving the diversity of the other organisms. A further implication is that understanding the drivers of microbial diversity will be central to predicting higher trophic level responses to environmental change, which is happening at marked rates in polar regions^44^.

The empirical SEM (Fig. 2a) incorporated all measured factors, including soil physicochemical properties that cannot be obtained through remote sensing. Therefore, an additional SEM was derived using only unstandardized coefficients associated with factors that are obtainable through remote sensing and GIS (e.g., wetness index, temperature, elevation, aspect, distance to the coast, and slope) (Supplementary Figure 3). In the future, this “predictive” SEM can be used to make spatially explicit predictions of biodiversity across the entire Dry Valley landscape. Ultimately, the model and its future version can be used to support the development of best management practices for this unique ecosystem protected by the Antarctic Treaty System (http://www.ats.aq).

In conclusion, we found that abiotic factors such as soil temperature and topography had important direct effects on richness as well as indirect effects mediated through physicochemical soil properties (Fig. 2a). However, contrary to our expectations, we also found that the correlations between the functional groups and spatial autocorrelation in the variation of the richness of the functional groups are a major determinant of the biological diversity of the system. This result suggests that biotic factors are an underestimated control on the complexity of the Dry Valley ecosystem and raises the question of whether biotic interactions and processes have been similarly underappreciated in other simple and/or extreme ecosystems. Furthermore, our findings highlight the fundamental importance of incorporating biotic factors and spatial constraints when forecasting community responses to changing environmental conditions. This has direct relevance to more complex ecosystems where biotic interactions play a markedly greater role in shaping community structure and ecosystem functioning.

## Methods

### Study area

Approximately 0.4% of Antarctica is permanently ice-free, and the main ice-free areas are the Antarctic Peninsula, the McMurdo Dry Valleys, and various mountains and nunataks along the Transantarctic Mountains^21^. Of these, the McMurdo Dry Valleys contain the largest contiguously ice-free areas (~4500 km^2^) and have been the focus of terrestrial biology research on the continent for the past 50 years^21,39^. The McMurdo Dry Valleys are situated in southern Victoria Land along the western coast of McMurdo Sound (between 160–164°E and 76–78°S) and contain markedly complex surface geology and topography that result in highly heterogeneous physicochemical conditions in soils across the landscape. The area chosen for this study comprises 220 km^2^ of largely ice-free terrain that includes Garwood, Marshall, and Miers Valleys as well as Shangri-La, an area west of Marshall Valley and enclosed by Joyce Glacier, Mt. Pams, and Mt. Lama (Fig. 1).

In addition to the extreme cold (mean annual air temperature of approximately −20 °C), the McMurdo Dry Valleys are characterized by strong winds, extreme aridity (precipitation of <10 cm per year water equivalent), and lack of appreciable solar input for much of the year^21^. Despite the extreme selective pressure, the Dry Valleys appear to sustain a functional but simple ecosystem comprised of prokaryotes, invertebrate fauna, and non-vascular flora^21^. Cyanobacteria (both aquatic and edaphic) appear to be the main primary producers, although important photosynthetic activity occurs in lithic communities (i.e., endoliths, hypoliths, and chasmoendoliths) as well as mosses and lichens^23^.

The invertebrate fauna consists of the microarthropods Collembola (i.e., springtails) and Acari (i.e., mites), as well as a range of microinvertebrates including nematodes, tardigrades, and rotifers. Nematodes are the dominant invertebrate taxon across much of the landscape, and their distribution and abundance primarily correlate with the presence of liquid water, pH, salinity, and inorganic carbon^45,46^. Taxonomic diversity for nematodes is low (five species), but abundances can be as high as hot desert soils^23,46^. Rotifers (four species) and tardigrades (eight species) are present but more restricted to ephemerally wetted areas^23,46^. A single species of Collembola (*Gomphiocephalus hodgsoni*) represents the largest (albeit only <1.4 mm in length) terrestrial animal in the Dry Valleys, whereas two species of Acari (*Stereotydeus mollis* and *Nanorchestes antarcticus*) are known within the region. The microarthropods share similar distributional patterns and are more commonly found in soils under rocks on stable and sunny slopes close to water sources. Soil microbial communities are composed of predominantly heterotrophic bacteria^27,47^ (archaeal abundance and diversity appear to be very limited^48^) and fungi^49^, and constitute by far the largest biomass in the ecosystem^21^.

### Tile delineation

Using a digital elevation model (DEM) based on LIDAR data for the area^50,51^, slope (in degrees), elevation (in meters above sea level), and aspect (N, S, E, and W) were generated as the primary inputs for the nzTABS GIS model. The GIS model also included geological (i.e., major bedrock lithologies) and geomorphological (i.e., fluvial, aeolian, and glacial) datasets from published sources^52–54^, augmented by analyses of ALOS, LandSat, and MODIS satellite imagery, aerial photographs, and subsequent field mapping (Table 2). Using the GIS model, the study area (excluding areas covered by ice, snow, and water) was divided into more than 600 geographically and geologically distinct polygons (hereinafter “tiles”, minimum 1.5 km^2^). Tile boundaries were delineated where the combination of topographic and geologic attributes changed (Table 2 & Supplementary Figure 4). Majority filtering was used to smooth spatial variability and avoid the creation of large numbers of small tiles unsuitable for sampling. On-the-ground assessments were carried out in November 2008 to confirm the reliability of delineations, and 554 tiles were chosen for sampling to encompass the entire range of geographical and geological heterogeneity (Fig. 1c) with replications for most common combinations of tile-defining characteristics. However, we acknowledge that there may be a systematic bias against habitats that cannot be accessed safely (e.g., steep scree slopes).

**Table 2:** Landscape-scale variables captured by nzTABS

Category	Variables
Remote Sensing and GIS (Satellite and LIDAR)	Elevation^a^ Slope^a^ Aspect^a^ Snow/Ice/Water Presence^a^ Distance to the Coast Soil Surface Temperature Wetness Index^60^
Geology	Bedrock Geology^a^^53^ Glacial Geomorphology^a^
Biology	Lichen and Moss (Abundance and Size) Endolith and Hypolith (Abundance) Cyanobacterial Mat (Abundance and Size) Invertebrates (Abundance and Taxonomy) ATP Level Bacterial Richness (ARISA) Cyanobacterial Richness (ARISA) Fungal Richness (ARISA)
Geochemistry	pH Conductivity Water Activity (*A*_w_) Total Soil Moisture Content Total Soil C & N

^a^Variables used for tile delineation

### Tile sampling

**Fig. 3:**
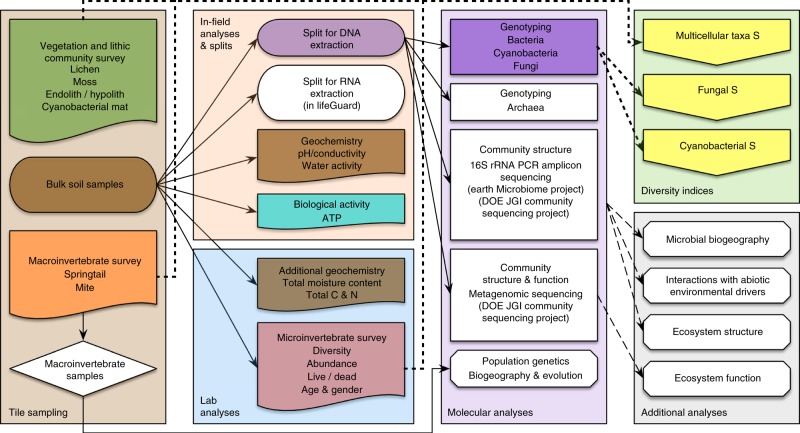
Flow diagram for nzTABS sample analysis. “S” represents the richness and composition of multicellular taxon and microbial assemblages. Solid lines represent transfer or utilization of physical samples (including DNA), and dashed lines represent analysis of information. Colored components are included in the present study

Sampling of soils and biological communities was carried out over two successive austral summers (January 2009 and January 2010). Within each tile, a sampling site was chosen based on feasibility (a safety consideration) as well as sampling route planning. Each sampling site had to be inside its corresponding tile and representative of the geographic and geologic attributes for the tile. At each sampling location (GPS coordinates and elevation were recorded), the top 10 cm (top 2 cm for prokaryotes) of soil was collected aseptically using a trowel from multiple spots within a 1 m^2^ area for the following subsamples (Fig. 3): bulk soil (~400 g) with large pebbles (>2 cm diameter) removed aseptically and homogenized in a sterile 42 oz. Whirl-Pak; soil (~20 g) for moisture content measurement, subsampled from homogenized bulk soil into a sterile 15 mL centrifuge tube sealed with Parafilm; soil (~300 g) for microinvertebrate count, stored in a sterile 18 oz. Whirl-Pak (pebbles not removed to minimize disturbance).

A microarthropod survey (i.e., springtails and mites) was carried out by examining the underside of small (5–10 cm dia.), flat (<2 cm thickness), and preferably dark rocks within a 20 m radius of the soil sampling location for 10 min^55^. The number and types of microarthropods observed were recorded, and the organisms were collected using an aspirator and preserved in a vial containing 100% ethanol for later analyses^55^. A survey of vegetation (i.e., lichens, mosses, algae, and cyanobacterial mats) and lithic communities (i.e., hypoliths and endoliths) was carried out along a transect (20 m long and 2 m wide, 40 m^2^) adjacent to the soil sampling location. Vegetation presence was recorded quantitatively in 100 cm^2^ units for each taxon, and the numbers of observed lithic communities were recorded. All our activities were conducted in accordance with the McMurdo Dry Valley Antarctic Specially Managed Area manual and were deemed to have limited and transient impact to the environment according to the Preliminary Environmental Evaluation from the New Zealand Ministry of Foreign Affairs and Trade. All soil samples collected are stored at −60 °C or −80 °C at the International Centre for Terrestrial Antarctic Research at the University of Waikato.

Soil samples were subsequently aliquoted and analyzed for total ATP, pH, conductivity, water activity (*A*_W_), total moisture content, microinvertebrate (i.e., nematodes, tardigrades, and rotifers) richness and abundance, and organic carbon and total nitrogen content (Fig. 3 and Supplementary Methods), as well as used for bulk DNA extraction (Supplementary Methods). All extracted DNA samples are available from the Antarctic Genetic Archive (AGAr, https://ictar.aq/antarctic-genetic-archive/) at the University of Waikato.

### DNA-based analysis of microbial communities

After quantification and quality check (Supplementary Methods), extracted DNA samples were used for molecular fingerprinting of bacterial (total and cyanobacteria-only) and fungal communities based on automated ribosomal intergenic spacer analysis (ARISA)^56–58^. Briefly, the intergenic spacer between the 16S and 23S rRNA genes of the bacterial/cyanobacterial ribosomal operon and the intergenic spacer between the 18S and 23S rRNA genes of the fungal ribosomal operon were amplified using PCR for each sample (Supplementary Methods).

ARISA fragment length profiles (Supplementary Data 1) were analyzed using an in-house pipeline (a combination of Applied Biosystem Peak Scanner and custom R and Python scripts, see Supplementary Data 2) that examines all peaks between 100 and 1200 base pairs for cyanobacterial electropherograms and 100 and 1400 base pairs for fungal electropherograms^57^. Peaks in these size ranges that made up greater than 0.3% of all peaks over 30 relative fluorescence units in each electropherogram were accepted as true peaks. The total number of true peaks was taken as a measure of taxon richness for each sample. Peaks within one base pair of one another were binned for the purpose of comparing electropherograms between samples.

ARISA was used to measure richness due to its proven ability to detect and discern diversity of edaphic cyanobacteria signals in the Dry Valleys over 16S rRNA gene PCR amplicons^27,57^ and its proven ability to capture fungal diversity patterns in Dry Valley soils in a consistent and cost-effective manner.

### Environmental metadata

A number of key environmental attributes were derived from satellite imagery and the DEM, including surface soil temperature, a topographically derived “wetness index”, and distance to the coast. Soil surface temperatures were obtained from Landsat 7 ETM+ using band 6 (at 60 m resolution), which captured the up-welling thermal infrared spectrum (in the 10.4–12.5 μm band). Landsat 7-derived temperature data corresponding to locations of forty-five on-the-ground temperature loggers (DS1921G iButtons, Maxim Integrated, San Jose, CA) were compared with records from the iButtons, and significant positive correlations between the two data sets were found^59^.

Wetness index, which produces a relative index of liquid water availability in summer, was calculated using a GIS-based model using variables that influence the volume and distribution of water. Remote sensing images from the Moderate Resolution Imaging Spectroradiometer (MODIS) sensor collected over several years were used to calculate an average index of snow cover, which was then combined with other water sources such as glaciers and lakes. This resulted in a probable water source model representing the highly heterogeneous distribution of water sources in the Dry Valleys^60^. The water source model was used to weight a hydrological flow accumulation model^61^ that used slope derived from LIDAR elevation data captured for most parts of the Dry Valleys^50^. These data were then used to calculate a Compound Topographic Index (CTI), a steady-state wetness index based on both slope and upstream contributing area^62^. CTI takes the form: ${\mathrm {CTI}} = {\mathrm{ln}}\left( {\frac{{A_s}}{{\tan \beta }}} \right)$ where *A*_s_ is the upslope contributing area in m^2^ per unit width orthogonal to the flow direction, and *ß* is the slope angle in radians^63^. The resultant model is a relative index of potential water availability, given the availability of melt water sources and topographical features.

Distance to the coast value was calculated as the Euclidean distance (in meters) from the sampling point to the closest point on coastline, which in turn was defined by cells with zero elevation in the DEM. Specifically, the shortest distance was determined by the perpendicular from the coastline to the sampling point. After quality control (removal of tiles with missing or questionable information, such as unintended duplicates and incorrect GPS location), data for 490 samples were included in the analysis.

### Data analysis

A broad suite of geological, geographical, geochemical, hydrological, and biological variables (Table 2 and Supplementary Data 3) were collected and evaluated to derive the most parsimonious set of predictors of biodiversity in our study area. Biodiversity is represented by the richness of key autotrophic and heterotrophic groups and the presence of known taxa. Specifically, species richness of cyanobacteria and fungi was estimated using the number of ribosomal intergenic spacer length-polymorphic fragments observed from community fingerprinting analyses. These intergenic spacers exhibit length polymorphism across species and even at the intra-species level, and the length profiles of PCR fragments are therefore indicative of the diversity and abundance of microbial communities. We note that these techniques do not resolve richness at a consistent taxonomic level; however, given that they can both over- and under-estimate species-level richness, we do not believe the results were influenced by systematic biases. Taxon richness for multicellular taxa was represented by the number of the following supraspecific taxa present in a sample: nematodes, rotifers, tardigrades, springtails, mites, cyanobacterial mats, mosses, lichens, and hypolithic consortia. These taxonomic groups also correspond to distinct trophic/functional groups in the system. Specifically, the animals are all primary consumers of both bacteria and fungi, cyanobacteria are the main primary producers besides mosses, and fungi represent the major microbial decomposer group. Given the very low number of metazoan (Supplementary Figure 1), cyanobacterial^57^, and fungal^49^ species in the Dry Valleys, relatively small increase in the species richness of each compartment may imply a marked increase in the complexity of the system in terms of increased number of ecological interactions.

To verify that inferences made from patterns in species richness apply similarly to community composition, richness was correlated with community composition in all three groups of organisms (i.e., cyanobacteria, fungi, and multicellular taxa) based on an analysis of *nestedness* (R script available upon request). Nestedness occurs when species-poor communities are generally subsets of species-rich communities, and when rare species tend to only occur in species-rich communities. The nestedness of each of the three community matrices was evaluated by calculating their respective “temperatures”, which determine whether species-poor communities are subsets of species-rich ones. The “temperatures” were calculated using the “nestedtemp” function^64^ in the “vegan” library of R^65^, and their significance was assessed via permutation using the “oecosimu” function. The relationship between richness and non-metric multidimensional scaling (NMS) ordinations (based on Bray–Curtis similarity) of community composition (obtained using the “metaMDS” function in “vegan”)^65^ was quantified using correlation analysis.

Overall, all these preliminary analyses supported the assumption that in the specific system analyzed in this work, species richness of major functional groups is the best metric to describe the richness of each group as well as the correlations between groups and the relationship between biota and abiotic factors.

Cyanobacterial richness, rather than total bacterial richness, was included in our analysis for the following reasons. First, including both would effectively be “double-counting” since total bacterial richness includes cyanobacteria as well. Second, cyanobacteria are arguably the most critical group of bacteria, given their large proportional input to primary production in this extreme environment. Finally, cyanobacterial richness was significantly and positively correlated with total bacterial richness (*r* = 0.31, *P* < 0.0001), so knowing the richness of one group provides reasonable estimates about the richness of the other.

Biological communities closer in space are likely to be more similar in species richness and community composition. However, historical population- and landscape-level processes that are relatively independent of environmental conditions can also be a driver of Antarctic biodiversity^13,20,66,67^. Thus, environmentally independent spatial variables were computed to account for spatial patterns linked to intrinsic population- and landscape-level processes, such as dispersal limitation or source-sink dynamics^8^ (see Supplementary Methods). Competition- and predation-related direct biotic interactions were not explicitly considered due to limited evidence for such interactions among Dry Valley biota^23,28^.

To represent spatial patterns driven by intrinsic population- and community-level processes (e.g., limited dispersal), environmentally independent spatial variables were obtained as follows. First, optimal (in terms of describing spatial autocorrelation) combinations of Principal Coordinates of Neighbor Matrices (PCNM) were calculated^8^. To explicitly model spatial patterns that are independent of environmental gradients, the PCNMs were regressed against all environmental variables to allow extraction of the residuals (aka “spatial residuals”). The “spatial residuals” were then used in a linear regression model to predict the three biotic richness variables, and their predicted values were derived. This was followed by a principal component analysis (PCA) on these predicted values, allowing spatial patterns to be summarized in the multivariate distribution of the three biotic richness variables. The first two components (“s1” and “s2”) accounted for 90% of the environmentally independent spatial patterns. Finally, the net effect of spatial variation (s1 + s2) was captured through the use of a composite variable (diamond shape)^30^. These two spatial vectors thus account for all the spatial variation that is not explainable in terms of measured biotic and abiotic variables. This variation also implicitly account for the effects of spatial variation in unmeasured variables, which contribute to autocorrelation in measured variables.

Structural equation modelling (SEM) with composite latent variables was used to determine the relative importance of abiotic conditions, biotic interactions, and spatial patterns due to population- and community-level processes. Based upon previous work known at the time the model was fit^22,29,66–68^, an *a priori* SEM of biodiversity was built, in which topographic properties and surface temperature (summer average) are mediated through the effects of soil properties and indirectly influence the richness of cyanobacteria (which positively correlates with total bacterial richness as described above), fungi, and multicellular taxa (Supplementary Figure 2). To identify variables to be included in the *a priori* model, the entire set of predictors was evaluated to determine which variables were most likely to be important for reasons of parsimony, thereby eliminating soil age, geology, soil C, and conductivity.

To derive a final model with good fit to the data from the *a priori* SEM, non-significant pathways were removed, and theoretically justifiable pathways were added that were deemed to be important through inspecting the residual covariance matrices and modification indices. The relationship between biotic richness and geology was analyzed using ANOVA, and the final SEM was found to explain more variation than geology alone. The “total effects” of each variable on each biotic variable were calculated (Supplementary Table 1), which provides an order to which factors are most important by taking into account both direct and indirect effects (total effects = direct + indirect effects; indirect effects = sum of the products along each pathway). Finally, composite variables were used to estimate the net effects of three constructs (abiotic environmental filters, spatial processes, and biotic interactions) on each of three biotic response variables (cyanobacterial, fungal, and multicellular taxon richness)^30^.

### Code availability

Python, R, and Mplus scripts used to analyze the data are available in Supplementary Data 2 and 4.

## Supplementary information

Supplementary Items

Description of Additional Supplementary Items

Supplementary Data 1

Supplementary Data 2

Supplementary Data 3

Supplementary Data 4

## Data Availability

The final environmental and biological datasets generated and analyzed during the current study are available as Supplementary Data 1 through 3.
