# Supplementary material for: Biotic interactions are an unexpected yet critical control on the complexity of an abiotically driven polar ecosystem
Source: Commun Biol. 2019 Feb 15;2:62. doi: 10.1038/s42003-018-0274-5 (PMC6377621; doi:10.1038/s42003-018-0274-5)
Supplement: Supplementary file 1 — Supplementary Items [file 42003_2018_274_MOESM1_ESM.pdf]

Supplementary Figure 1: Trophic layers of the Antarctic Dry Valley ecosystem (adapted from Cary *et al*, 2010)

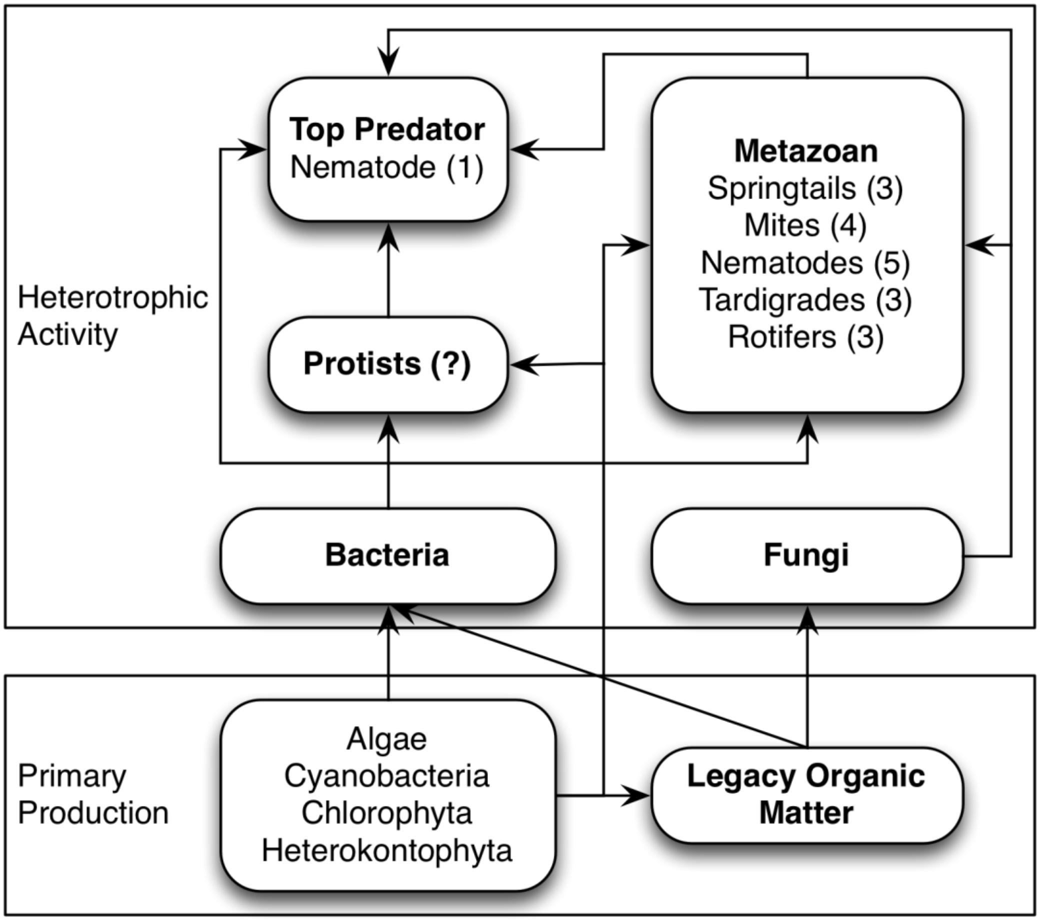

The number of species known for each taxon is denoted in parentheses. Cyanobacteria are the dominant primary producer, and cyanobacterial detritus associated with historic lacustrine features (i.e., legacy organic matter) remains an important input for heterotrophic microorganisms (Adams *et al.* 2006; Barrett *et al.* 2006). The top predators in the Dry Valley soil ecosystem are *Eudorylaimus antarcticus*, an omnivorous nematode, and *Scottnema lindsayae*, a bacterivorous nematode.

Supplementary Figure 2: Initial *a priori* graph-theoretic model

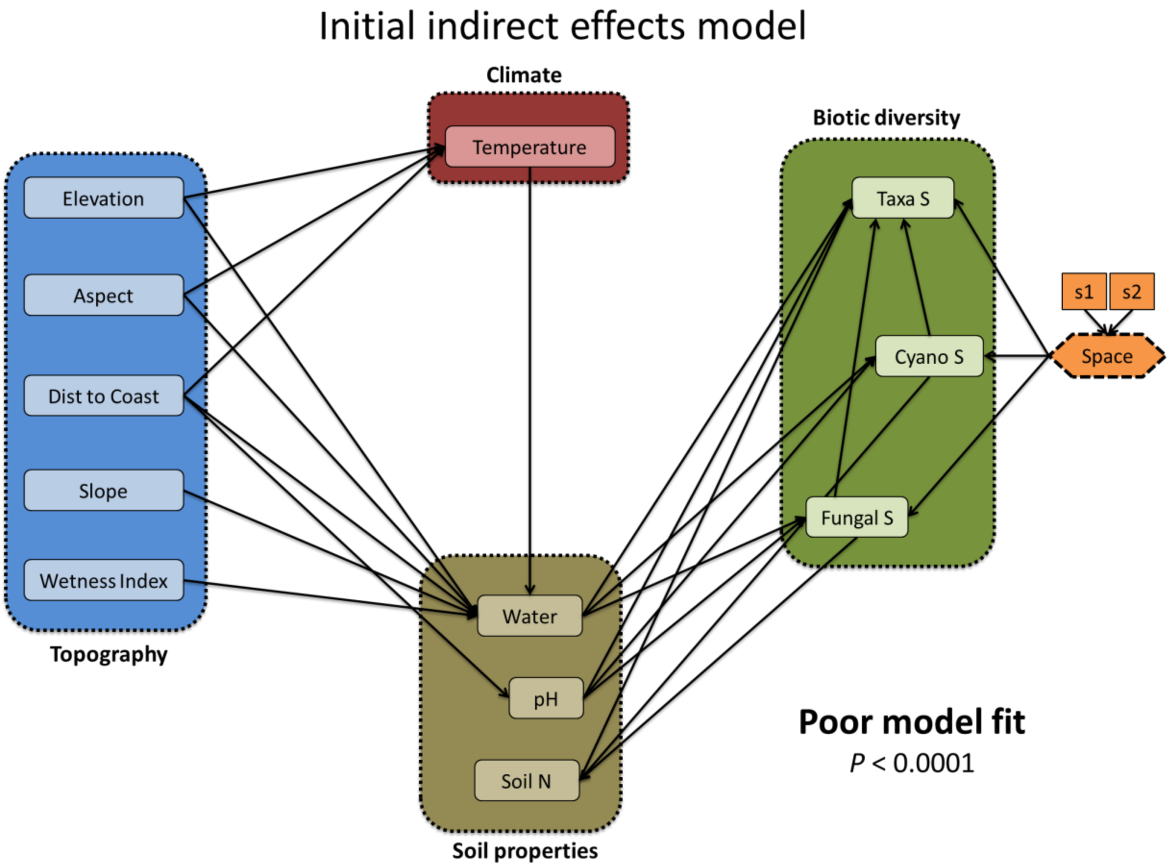

Topography and climate were hypothesized to indirectly affect biodiversity through the mediating effects of soil properties in this *a priori* model.

Supplementary Figure 3: "Predictive" structural equation model

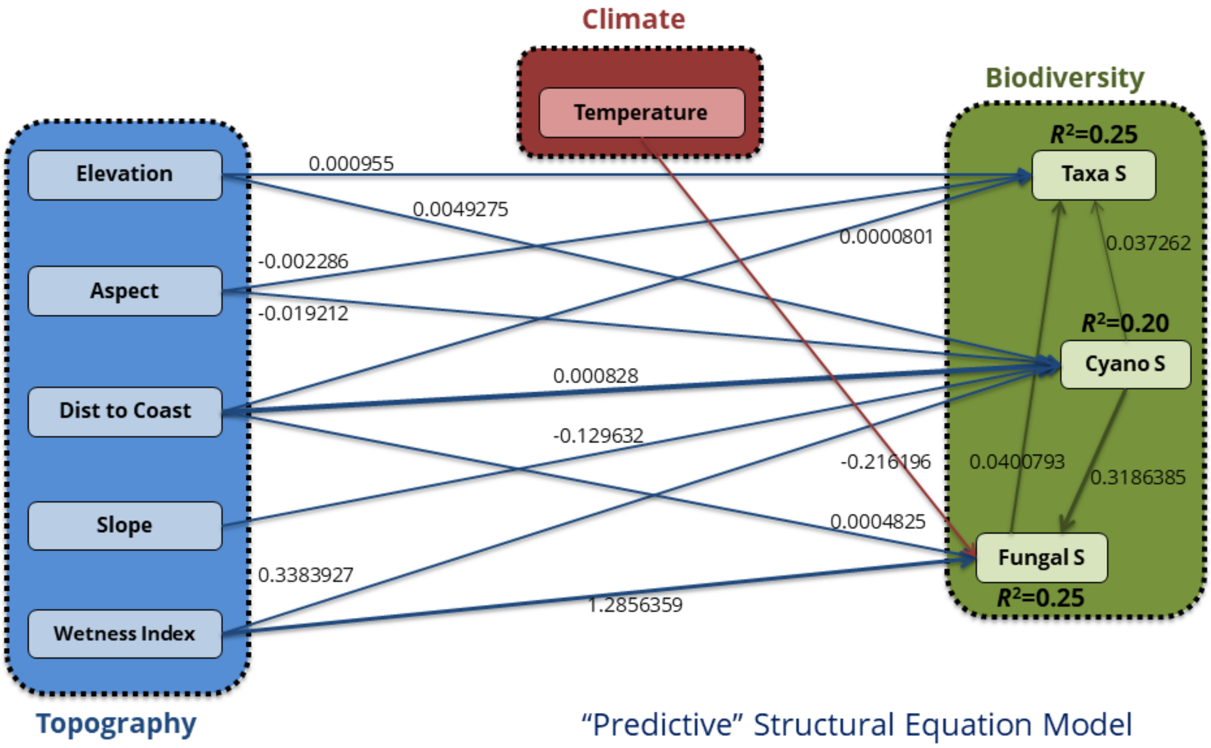

Predictive equations (including intercepts):

CYANO.S = 5.4838472 + 0.0049275*ELEV – 0.129632*SLOPE – 0.019212*ASPECT + 0.000828*COASTDIS + 0.3383927*WETNESS

FUNGAL.S = -0.090768 – 0.216196*TEMP + 0.0004825*COASTDIS + 1.2856359*WETNESS + 0.3186385*CYANO.S

TAXA.S = 0.6690415 + 0.000955*ELEV10 - 0.002286*DEGFN + 0.0000801*COASTDIS + 0.0400793*FUNGAL.S + 0.037262*CYANO.S

This model incorporates only unstandardized coefficients associated with factors obtainable through remote sensing and GIS.

Supplementary Figure 4: Example of tile delineation

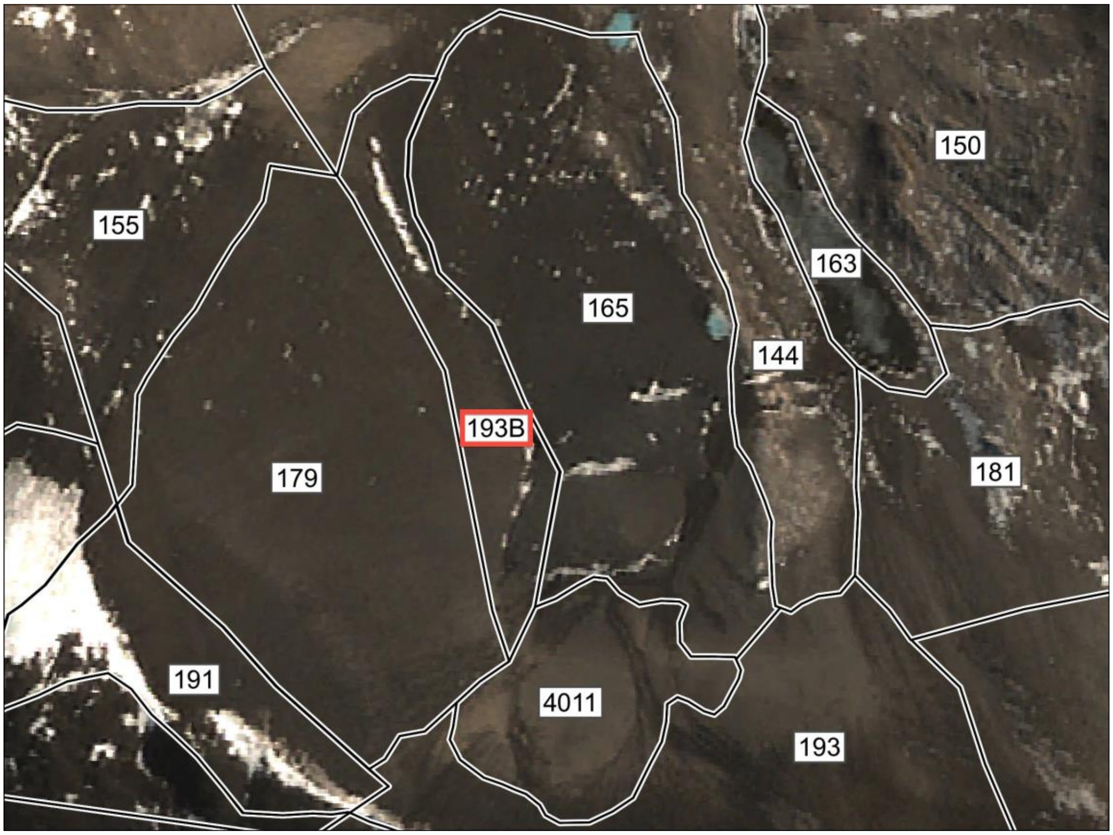

Tile boundaries were defined by where change occurred in one of the following topographical or geological properties: elevation, slope (average), aspect (average), and bedrock geology. For example, tile #193B is separated from #179 by elevation (600-800 m vs. 800+ m); from #4011 by geology (granite vs. aeolian), elevation (600-800 m vs. 800+ m), and aspect (E vs. N); from #165 by geology (granite vs. M1), aspect (E vs. flat), and slope (20+° vs. 0°); and from #144 by geology (granite vs. marble) and slope (20+° vs. 0-20°).

Supplementary Table 1: Total and indirect standardized coefficients (all are significant, P<0.05)

Response	Predictor	Total Effects	Indirect Effects	Direct Effects
Multicellular Taxa S	Elevation	0.20	0.08	0.12
	Aspect	-0.11	-0.02	-0.09
	Distance to the Coast	0.27	0.11	0.16
	Slope	-0.05	-0.05	
	Wetness Index	0.06	0.06	
	Temperature	-0.02	-0.02	
	Soil Moisture	0.06	0.06	
	pH	-0.02	-0.02	
	Soil N	0.25		0.25
	Cyanobacterial S	0.14	0.03	0.11
	Fungal S	0.15		0.15
	Spatial Variable	-0.21		-0.21
Cyano S	Elevation	0.16		0.16
	Aspect	-0.13		-0.13
	Distance to the Coast	0.33		0.33
	Slope	-0.11		-0.11
	Wetness Index	0.09		0.09
	Spatial Variable	0.40		0.40
Fungal S	Elevation	0.10	0.10	
	Aspect	-0.03	-0.03	
	Distance to the Coast	0.24	0.08	0.16
	Slope	-0.05	-0.05	
	Wetness Index	0.30	0.06	0.24
	Temperature	-0.12	-0.04	-0.08
	Soil Moisture	0.18	0.02	0.16
	pH	-0.10		-0.10
	Cyanobacterial S	0.20		0.20
	Spatial Variable	-0.34		-0.34

“S” represents the richness and composition of multicellular taxa and microbial assemblages.

SUPPLEMENTARY METHODS

Analysis of Soil Samples (Field). In the field laboratory, bulk soil samples were further aliquoted and processed for additional analyses:

1. DNA-based analyses (~80 g soil): aliquoted from homogenized bulk soil sample into a sterile 50 mL centrifuge tube. Analyses were carried out at the University of Waikato.
2. Soil total ATP analysis (100 mg soil x 2): aliquoted from homogenized bulk soil sample into sterile ATP assay tubes.
3. Soil geochemistry (~80 g soil): aliquoted from homogenized bulk soil sample through a 2 mm sieve into a 4 oz. Whirl-Pak. Analyses were carried out at Virginia Tech, Blacksburg, VA, USA.
4. Soil pH and conductivity (2 mL volume): aliquoted from homogenized bulk soil sample through a 2 mm sieve into a 15 mL centrifuge tube.
5. Soil water activity (A_w) (~15 g): aliquoted from homogenized bulk soil sample through a 2 mm sieve into an A_w measurement cup.

Total soil ATP was measured in duplicates (triplicates where the duplicates did not match) using a 3M Clean-Trace Beverage Test Kit (Acorn Scientific, Auckland, NZ) with a modified protocol. In short, 100 µL of Extractant Buffer was added to 100 mg of soil and allowed to incubate for 60 seconds. 75 µL of ATP Assay Solution was then added to the sample, which was immediately read using a 3M Clean-Trace NG Luminometer (Acorn Scientific). Total soil ATP levels were recorded as relative fluorescence units, and pure ATP solutions were used to check for signs of inhibition in samples with low readings. Soil pH and conductivity were measured using the slurry method (Lee *et al.* 2012). In brief, 10 mL of deionised water was added to a soil aliquot (2 mL) and mixed thoroughly. The pH and conductivity of the resulting slurry was

measured using a Thermo Scientific Orion 4-Star Plus pH/Conductivity Meter (Thermo Scientific, Auckland, NZ). Soil water activity (A_w) was measured using an AquaLab Pawkit Water Activity Meter (Decagon Devices Inc., Pullman, WA).

Analysis of Soil Samples (Laboratory). Soil samples for moisture content and microinvertebrate analyses were transported to McMurdo Station within 72 hours of collection and analyzed at the Crary Laboratory. Soil total moisture content was determined gravimetrically by the mass loss of soil heated to 105°C for 48 hours and recorded as percentage moisture content (Barrett *et al.* 2004). Microinvertebrates (i.e., nematodes, tardigrades and rotifers) were extracted from soils using a modified sugar-centrifugation technique (Freckman and Virginia 1997) and identified and enumerated using bright-field microscopy (Olympus CK40 Inverted Microscope, Olympus America Inc., Center Valley, PA). Population abundances were recorded as numbers of individuals per kg soil, corrected to oven-dry weight equivalent. Demographic information for nematode populations (i.e., gender, juvenile/adult, alive/dead) was also recorded but not used for the construction of the SEM. Observed protozoan (i.e., flagellates, amoebae, and ciliates) abundances were recorded, but the data were not included in the SEM since reliable characterizations of protozoan abundance and diversity greatly exceeded our logistical capability (Bamforth *et al.* 2005).

Soil samples for DNA-based analyses were transported to Scott Base within 72 hours of collection, where they were stored at -20°C. They were then shipped to University of Waikato under refrigerated conditions and stored at -80°C until analyzed. DNA was extracted from soils and analyzed as described below. A subsample of soil was air-dried and ground in a ball mill to a fine homogenous powder for geochemical analyses. Organic carbon and total nitrogen (inorganic

and organic) content was determined from a 300 mg acidified aliquot of this dried, homogenized material with a CE Elantech Flash EA 1112 Elemental Analyzer (Lakewood, NJ) at the Virginia Tech Ecosystem Analysis Laboratory (Barrett *et al.* 2009).

DNA Extraction and Characterization. For each sample, 0.7 g of soil was added to a microcentrifuge tube containing 0.5 g each of 0.1 mm and 2.5 mm silica-zirconia beads (BioSpec Products, Bartlesville, OK, USA). 270 µL phosphate buffer (100 mM NaH_2PO_4) and 270 µL SDS lysis buffer (100 mM NaCl, 500 mM Tris pH 8.0, and 10% SDS) were added, and samples were bead-beaten for 10 minutes on a Vortex Genie 2 with a 24-tube vortex adapter (Mo Bio Laboratories Inc., Carlsbad, CA, USA). 180 µL CTAB extraction buffer (100 mM Tris-HCl, 1.4 M NaCl, 20 mM EDTA, 2% CTAB, 1% PVP, 0.4% BME) was then added, and samples were shaken at 300 rpm and 60 °C for 30 minutes. Samples were centrifuged at 16,000 g for 3 minutes, prior to the addition of 350 µL chloroform:isoamyl alcohol (24:1) and 35 µL 10 M ammonium acetate. Samples were vortexed and centrifuged at 16,000 g for 5 minutes. A 200 µL aliquot of the aqueous phase of each sample was transferred to a 96 well lysis block and processed using an X-tractor Gene liquid handling robot (Corbett Life Sciences, Concorde, NSW, Australia), using the DX Universal liquid sample DNA Extraction Protocol (CorProtocol No. 14104 Version 02). Samples were eluted in 80 µL TE pH 8.5 (10 mM Tris-HCl, 0.5 mM EDTA). Negative controls, consisting of bead tubes with no sample added, were processed as described above and included in each lane of the lysis block to assess potential contamination of extracts.

For samples yielding DNA concentrations less than 1.8 ng/µL, extractions were repeated manually, without processing on the X-tractor Gene, to increase yields. The lysis steps were

completed as outlined above, and the method after the chloroform step was modified as follows. The final ammonium acetate concentration of the lysate was brought to 2.5 M, 300 µL chloroform:isoamyl alcohol (24:1) was added, samples were vortexed, and centrifuged at 16,000 g for 5 minutes. The entire aqueous phase was transferred to a new tube and the chloroform step repeated with an equal volume of chloroform:isoamyl alcohol (24:1). The aqueous phase was transferred to a new tube and DNA was precipitated with addition of 0.54 volumes of isopropanol followed by centrifugation at 16,000 g for 20 min. Pellets were washed by adding 1 mL 70 % ethanol, centrifuged at 16,000 g for 5 min, and the supernatant discarded. Dried pellets were resuspended in 30 µL TE pH 8.5.

DNA extracts were quantified using Quant-iT Picogreen dsDNA reagent (Invitrogen, Auckland, New Zealand) on a FLUOstar optima fluorescence plate reader (BMG Laboratories, Offenburg, Germany). Briefly, 100 µL of picogreen solution (picogreen diluted 1:200 in TE) was added to each well of a black 96 well plate, containing 95 µL TE and 5 µL sample or standard containing 0 to 25 ng/µL lambda dsDNA (Invitrogen). Samples were excited at 485 nm and emission was measured at 520 nm. All extracts with DNA concentrations exceeding 2.2 ng/µL, were adjusted to between 1.8 and 2.2 ng/µL in TE to ensure consistent template concentrations in community analyses. The high-throughput DNA extraction method yielded >1.8 ng/µL for 386 samples while the manual adaptation of the method yielded >1.8 ng/µL for a further 87 samples. For the remaining 17 samples, DNA yields were <1.8 ng/µL but were sufficient for PCR amplification. DNA was below limits of detection in all negative controls.

Molecular Analysis of Microbial Communities Using ARISA. PCR targeting the intergenic spacer between the 16S and 23S rRNA genes of the cyanobacterial ribosomal operon and the

intergenic spacer between the 18S and 23S rRNA genes of the fungal ribosomal operon were completed for each extraction. Primer pairs for cyanobacterial amplifications were CY-ARISA-F (Wood *et al.* 2008) (5'-PET-GYC AYR CCC GAA GTC RTT AC-3') (Applied Biosystems) and 23S30R (Wood *et al.* 2008) (5'-CHT CGC CTC TGT GTG CCW AGG T-3') (Integrated DNA Technologies) and for fungal amplifications were ITS1F (Gardes and Bruns 1993) (5'-FAM-CTT GGT CAT TTA GAG GAA GTA A-3') and 3126T (Sequerra *et al.* 1997) (5'-ATA TGC TTA AGT TCA GCG GGT-3') (Integrated DNA Technologies). Each 25 µL reaction contained 1X PCR buffer, 3 mM $MgCl_2$, 1 unit of Platinum Taq DNA Polymerase (Invitrogen), 0.25 µM primers, 0.2 mM dNTPs (Invitrogen), and 5 µL of template DNA. Thermal cycling was completed on a Bio-Rad DNA Engine Peltier Thermal Cycler 200 (Bio-Rad, Hercules, CA, USA). Thermocycling conditions for cyanobacterial ARISA consisted of 94 °C for 2 min; 35 cycles of 94 °C for 20 s, 55 °C for 15 s, and 72 °C for 1 min 30 s; and 72 °C for 3 min. Thermocycling conditions for fungal ARISA consisted of 94 °C for 3 min; 35 cycles of 94 °C for 20 s, 52 °C for 20 s, and 72 °C for 1 min 15 s; and 72 °C for 5 min. Amplicons were diluted 1:20 in de-ionized water. A mixture containing 2 µL of diluted amplicon, 0.13 µL of Liz-1200 internal size standard (Applied Biosystems), and 7.87 µL of HiDi formamide (Applied Biosystems) was heat denatured at 95 °C for 5 minutes and cooled to 4 °C for 2 minutes, before being resolved on an ABI 3130 Genetic Analyzer (Applied Biosystems) at the University of Waikato DNA Sequencing Facility.

Literature Cited

Adams, B. J., R. D. Bardgett, E. Ayres, D. H. Wall, J. M. Aislabie, S. Bamforth, R. Bargagli, S. C. Cary, P. Cavacini, L. Connell, P. Convey, J. Fell, F. Frati, I. D. Hogg, K. Newsham, A.

O'Donnell, N. Russell, R. Seppelt, and M. I. Stevens. 2006. Diversity and distribution of Victoria Land biota. Soil Biology and Biochemistry 38:3003–3018.

Bamforth, S. S., D. H. Wall, and R. A. Virginia. 2005. Distribution and diversity of soil protozoa in the McMurdo Dry Valleys of Antarctica. Polar Biology 28:756–762.

Barrett, J. E., M. N. Gooseff, and C. Takacs-Vesbach. 2009. Spatial variation in soil active-layer geochemistry across hydrologic margins in polar desert ecosystems. Hydrology and Earth System Sciences 13:2349.

Barrett, J. E., R. A. Virginia, D. H. Wall, A. Parsons, L. Powers, and M. Burkins. 2004. Variation in biogeochemistry and soil biodiversity across spatial scales in a polar desert ecosystem. Ecology 85:3105–3118.

Barrett, J. E., R. A. Virginia, D. W. Hopkins, J. M. Aislabie, R. Bargagli, J. G. Bockheim, I. Campbell, W. B. Lyons, D. L. Moorhead, and J. Nkem. 2006. Terrestrial ecosystem processes of Victoria Land, Antarctica. Soil Biology and Biochemistry 38:3019–3034.

Freckman, D., and R. A. Virginia. 1997. Low-diversity Antarctic soil nematode communities: distribution and response to disturbance. Ecology 78:363–369.

Gardes, M., and T. D. Bruns. 1993. ITS primers with enhanced specificity for basidiomycetes--application to the identification of mycorrhizae and rusts. Molecular Ecology 2:113–118.

Lee, C. K.-W., B. A. Barbier, E. M. Bottos, I. R. McDonald, and S. C. Cary. 2012. The Inter-Valley Soil Comparative Survey: the ecology of Dry Valley edaphic microbial communities. The ISME Journal 6:1046–1057.

Sequerra, J., R. Marmeisse, G. Valla, P. Normand, A. Capellano, and A. Moiroud. 1997. Taxonomic position and intraspecific variability of the nodule forming *Penicillium nodositatum* inferred from RFLP analysis of the ribosomal intergenic spacer and random

amplified polymorphic DNA. Mycological Research 101:465–472.

Wood, S. A., A. Rueckert, D. A. Cowan, and S. C. Cary. 2008. Sources of edaphic cyanobacterial diversity in the Dry Valleys of Eastern Antarctica. The ISME Journal 2:308–320.
