# Supplementary material for: Biotic interactions are an unexpected yet critical control on the complexity of an abiotically driven polar ecosystem
Source: Commun Biol. 2019 Feb 15;2:62. doi: 10.1038/s42003-018-0274-5 (PMC6377621; doi:10.1038/s42003-018-0274-5)
Supplement: Supplementary file 2 — Description of Additional Supplementary Items [file 42003_2018_274_MOESM2_ESM.docx]

**Description of Additional Supplementary Files**

**File Name**: Supplementary Data 1

**Description**: Cyanobacterial and fungal ARISA peak profiles

- CyanobacterialARISA.txt:

Cyanobacterial ARISA peak profile of all samples exported from Peak Scanner and concatenated. To be used with the genotyping pipeline (Supplementary Data 2).

- FungalARISA.txt:

Fungal ARISA peak profile of all samples exported from Peak Scanner and concatenated. To be used with the genotyping pipeline (Supplementary Data 2).

**File Name**: Supplementary Data 2

**Description**: Genotyping pipeline for processing ARISA peak profiles

- Herbold_tRFLP_Parse_ABI_V5.py:

Python script for processing concatenated peak profiles (requires the R script below).

- Herbold_FilteringandBinning_V5.R:

R script for filtering and binning peak profiles, used as part of the Python script.

- ReadMe_V5_CKL.txt:

User manual for the genotyping pipeline.

- DemoData.txt:

A test dataset to demo the code.

- ClusBinMatrix_G_DemoData.txt_LogNorm_alpha_0.001_n200_cdivisive_b1_r4_s50_l0 .001_m20_x1200.txt:

The primary expected output from the test dataset.

**File Name**: Supplementary Data 3

**Description**: Master data spreadsheet for nzTABS

- nzTABS_MasterData_2011-10-31.xlsx:

The complete geographic, topographical, geological, environmental, geochemical, and biological (both observational and molecular genetic) data collected for Miers, Marshall, and Garwood Valleys through the New Zealand Terrestrial Antarctic Biocomplexity Survey.

- nzTABS_MasterData_2011-10-31_ReadMe.docx:

Detailed descriptions of all the fields in nzTABS_MasterData_2011-10-31.xlsx

**File Name**: Supplementary Data 4

**Description**: Code for calculating environmentally independent spatial variables and constructing structural equation models

- SpatialVectorScript.R:

R script for calculating spatial vectors that account for spatial variation not explainable in terms of measured biotic and abiotic variables.

- nzTABS.dat:

Test dataset for the spatial vector analysis R script.

- ReadMe_TC.txt:

User manual for the spatial vector analysis R script.

- nzTABS_SpaceResid_5_Composites.inp:

Test input file for the Mplus SEM code.

- nzTABS_SEM_dataset_SpaceResid.dat:

Test sample file for the Mplus SEM code.

- ReadMe_DCL.txt:

User manual for the Mplus SEM code.

- nzTABS_SEM_Mplus_Code.txt:

Mplus code and results (including standard errors associated with each of the pathways) for the construction and evaluation of the final structural equation model
